# Leukostasis and pigment epithelium-derived factor in rat models of diabetic retinopathy

**Published:** 2007-06-29

**Authors:** Masato Matsuoka, Nahoko Ogata, Keizo Minamino, Miyo Matsumura

**Affiliations:** Department of Ophthalmology, Kansai Medical University, Osaka, Japan

## Abstract

**Purpose:**

Spontaneously diabetic Torii (SDT) rats, an animal model of type 2 diabetes, have a low incidence of neovascular formation and an absence of non-perfused areas in their retinas at the proliferative stage that presents tractional retinal detachment with fibrous proliferation. The aim of this study was to determine whether leukostasis is present in the retina, to evaluate the levels of pigment epithelium-derived factor (PEDF) and intracellular adhesion molecule-1 (ICAM-1) levels in the blood of SDT rats, and to examine the effects of PEDF on leukostasis.

**Methods:**

SDT rats, streptozotocin-induced diabetic (STZ) rats, and control Sprague-Dawley (SD) rats were studied. The index of leukostasis in the retina was determined immunohistochemically by counting the number of labeled adherent leukocytes. The levels of PEDF and the soluble intracellular adhesion molecule (sICAM)-1 in the plasma were measured. To investigate the effect of PEDF and vascular endothelial growth factor (VEGF) on leukostasis, the adhesion of monocytes to human umbilical vein endothelial cells (HUVECs) was assayed in vitro.

**Results:**

SDT and STZ diabetic rats showed a significant increase of retinal leukostasis compared to that of control SD rats, but SDT rats had noteworthy lower levels of leukostasis than STZ rats in long term experiments. The sICAM-1 levels and PEDF expression were up-regulated in both STZ and SDT rats, but the SDT rats showed significantly higher levels of PEDF than STZ rats. In vitro studies showed that exposure of HUVECs to VEGF increased the number of adhering monocytes, and PEDF inhibited the VEGF-induced leukostasis in a dose-dependent manner.

**Conclusions:**

The inhibition of the VEGF-induced leukostasis by PEDF is most likely responsible for the low incidence of capillary occlusion and retinal neovascularization in SDT rats.

## Introduction

Spontaneously diabetic Torii (SDT) rats are models of type 2 diabetes [1]. They develop hyperglycemia and glycosuria spontaneously at about 20-weeks-of-age but can survive for a long time without insulin treatment. More important, SDT rats exhibit severe ocular complications, such as tractional retinal detachment, which resembles proliferative diabetic retinopathy (PDR) in humans.

We recently reported that neovascularization of retinal vessels was present in some SDT rats with tractional retinal detachments and proliferative tissues, but the incidence was low. Non-perfused areas that indicated the presence of ischemic areas of the retina were not detected at all ages [2]. The low incidence of neovascular formation and absence of capillary occlusion in the retina make the SDT rat model different from the findings in typical PDR in humans. Interestingly, the expressions of pigment epithelium-derived factor (PEDF), a strong inhibitor of ocular angiogenesis, and vascular endothelial growth factor (VEGF), a strong stimulator of angiogenesis, were both up-regulated in the SDT rat retinas [2]. These findings are different from the low levels of PEDF in human eyes with diabetic retinopathy or in eyes with ischemia-induced retinal neovascularization [3-9].

Diabetic retinal neovascularization is considered to be a result of retinal ischemia caused by capillary occlusion. Leukocytic activation and the adhesion of leukocytes to the vascular endothelial cells initiate capillary occlusion in diabetic retinopathy [10,11]. Strong interactions between leukocytes and endothelial cells are governed by intracellular adhesion molecule (ICAM)-1 [12,13]. Increased levels of circulating soluble ICAM-1 (sICAM-1) are considered to be a risk factor for obstructive cardiovascular disease [14-16] and also for the progression of diabetic retinopathy [17].

We have shown that levels of both PEDF and VEGF in the retina of SDT rat are elevated [2]. Thus, the aim of this study was to determine the levels of PEDF and sICAM-1 in the blood of SDT rats, and to examine the effects of PEDF on the adhesion of leukocytes to endothelial cells.

## Methods

### Animals

The Committee of Animal Use of the Kansai University Medical School approved the experimental protocol, which also followed the ARVO guidelines for care and use of animals in eye research.

SDT rats (Tobacco Inc., Toxicology Research Laboratories, Kanagawa, Japan) and streptozotocin-induced diabetic rats (STZ) [11,18] were used. Normal Sprague Dawley (SD) rats were used as controls. SDT rats were defined as having diabetes if a dipstick test (Bayer Co., Tokyo, Japan) showed their urinary glucose level to be 3+ or higher. None of the SDT rats required insulin treatment. Diabetes was induced in 20 week-old SD rats with a single 60 mg/kg intraperitoneal injection of streptozotocin (STZ, Sigma, St. Louis, MO) [11,18]. Animals whose blood glucose levels were greater than 250 mg/dl at 48 h after the streptozotocin injection were classified as being diabetic. All animals were maintained in standardized housing with free access to water and standard laboratory chow.

### Body weight and blood samples

Each rat's body weight, blood glucose level, and glycosuria were measured at regular intervals. Two study periods were selected; 24 weeks for the short term effects of diabetes mellitus (DM), and 40 weeks for the long term effects. We used 8 eyes of 8 rats in each group at each study period in the experiments. At the end of every study period, blood samples were collected from each rat's jugular vein before the animal was euthanized. The plasma glucose level was measured by the glucose oxidase method using Glucose-Test Wako® (Wako Pure Chemical Co., Osaka, Japan).

### Index of retinal leukostasis

The retinal vasculature and adherent leukocytes were labeled with FITC-conjugated concanavalin A lectin (Con A: Vector Labs, Burlingame, CA) using a slight modification of a published perfusion-labeling technique [11,18,19]. Briefly, rats were anesthetized with an overdose of ketamine (50 mg/kg) intraperitoneally, then the rats were perfused with 250 ml/kg body weight of phosphate buffered saline (PBS) over 2 min to remove erythrocytes and non-adherent leucocytes. The eyes were enucleated and fixed in 2% paraformaldehyde for 1 h. Retinas were isolated, and flat mounts were prepared using an anti-fading fluorescence medium (Southern Biotechnology, Birmingham, AL). The retinal vessels were then examined with a fluorescence microscope (BX50, Olympus, Tokyo, Japan). Leukocytes in the retinal arterioles, venules, and capillaries were counted by two masked observers, and the total was used as an index of retinal leukostasis.

To confirm that the cells adhering to the vessels were leukocytes, were performed immunofluorescence with CD45, a leukocytes markers. After the adherent leukocytes were labeled with FITC-conjugated Con A, the flat-mounted retinas were permeabilized in 0.5% Triton X-100 (Sigma) in PBS for 24 h, then incubated overnight at 4 °C with a phycoerythrin-conjugated mouse monoclonal antibody against rat CD45 (1:200 dilution; Chemicon International Inc., Temecula, CA). The tissues were mounted, and the retinas were examined as described in the previous paragraph.

### Pigment epithelium-derived factor expression in plasma

PEDF levels in the plasma were measured by Western blot analysis from collected blood samples as described [2]. Band intensity was analyzed by gel plotting macro (NIH image Version 1.62, Mac OS9).

### Soluble intracellular adhesion molecule-1 levels in plasma

Plasma sICAM-1 levels were measured by an ELISA Kit (Quantkine® ICAM-1 ELISA Kit, R&D systems Inc., Minneapolis, MN), according to the manufacturer's protocol.

### Monocyte adhesion assay

Human monocytes were isolated from fresh, heparinized blood by density centrifugation using Ficoll-Paque Plus (Amersham Biosciences, Baied'Urfe, Quebec, Canada) according to the manufacturer's protocol. The blood was collected from healthy volunteers after we obtained informed consent. A published technique for calcein-AM-labeling (Dojindo Lab., Kumamoto, Japan) was used with slight modification [20,21]. The viability of the monocytes was assessed by the trypan blue exclusion test and was always >90%.

Human umbilical vein endothelial cells (HUVECs: Dainippon Pharmaceutical Co., LTD., Osaka, Japan) grown to confluence on collagen type I coated culture slides (BD Biosciences), were treated with VEGF_165_ (20 ng/ml in the medium, R&D Systems Inc.) as well as PEDF (0, 10, and 100 ng/ml in the medium, Chemicon International, Temecula, CA) for 24 h. Following the treatment, calcein AM-labeled monocytes (1.5x10^6^/ml, 200 μl/well) were added to the HUVECs and incubated for 60 min at 37 °C. Immediately before the assay, non-adherent cells were removed by washing four times with RPMI-1640 medium (Cat No. R8753, Sigma-Aldrich Japan K.K., Tokyo, Japan). The cells were then fixed for 30 min in 3.7% formaldehyde neutral buffer solution (Sigma-Aldrich Japan K.K., Tokyo, Japan) for 30 min at room temperature. The adherent monocytes were labeled with rhodamine-conjugated concanavalin A lectin (40 μg/ml in PBS, pH7.4, Vector Labs). The images were captured with a fluorescence microscope and images were captured in digital format.

### Statistical analyses

The results were expressed as mean±standard error of the means (SEM), and the data were analyzed using the one way analysis of variance (ANOVA) with Fisher's the least significant difference (LSD) when comparing groups. A p value of <0.05 was accepted as significant.

## Results

### Index of retinal leukostasis

**Figure 1:**
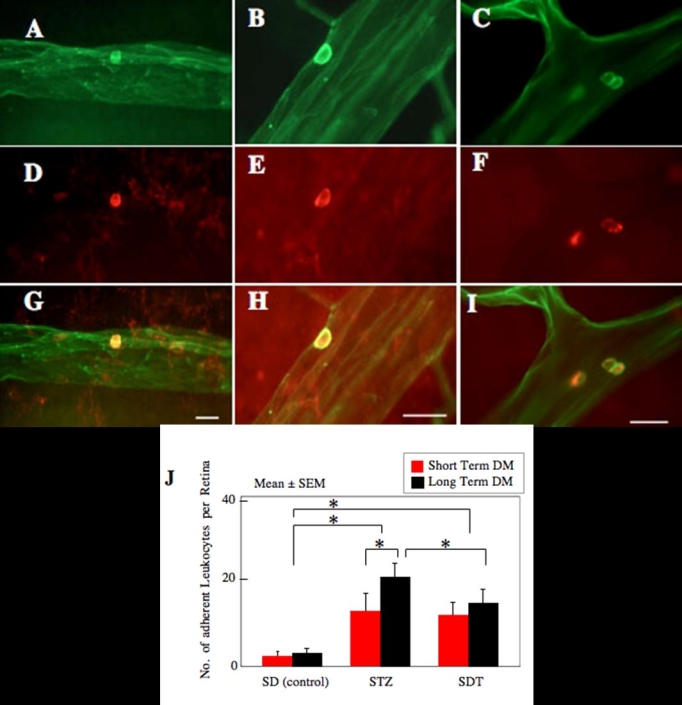
Adherent leukocytes in the retinal vasculature. Confocal fluorescence photographs of retinal vasculature and adherent leukocytes in Sprague-Dawley (SD) rats as control (**A**, **D**, **G**), streptozotocin-induced diabetic rats (STZ), which were spontaneously diabetic Torii (SDT) rats: (**B**, **E**, **H**), and SDT rats (**C**, **F**, **I**). The retinal vasculature and adherent leukocytes were stained green by FITC-conjugated concanavalin A lectin (**A**, **B**, **C**), and the adherent leukocytes were labeled with phycoerythrin-conjugated anti-rat CD45 antibody (**D**, **E**, **F**). The adherent leukocytes appear yellow in the merges images (**G**, **H**, **I**). Scale bar equals 20 μm. **J** shows the Index of retinal leukostasis. The adherent leukocytes were significantly increased in STZ rats compared to that of control SD rats in short term experiments (control vs STZ, p<0.05), and in long term experiments (p<0.05). SDT rats in short term experiments (p<0.05), and in long term experiments (p<0.05) also showed significantly increased adherent leukocytes compared to that of controls. However, SDT in long term experiments show significantly lower levels of leukostasis compared to that of STZ rats. In addition, the adherent leukocytes were significantly increased with duration of diabetes in STZ rats (p<0.05), but SDT rats did not show the increase with duration of diabetes. The data were analyzed by ANOVA. Asterisk equals p<0.05. DM represents diabetes mellitus. Eight eyes are used in each group at each study period.

Leukocytes adhering to the retinal vessels in control SD rats (Figure 1A,D,G), STZ rats (Figure 1B,E,H), and in SDT rats (Figure 1C,F,I) were counted while being viewed in whole mounts of the retina under confocal fluorescence microscopy. The retinal vasculature and adhering leukocytes were labeled green with FITC-conjugated concanavalin A lectin (Figure 1A-C), and the leukocytes were labeled red with phycoerythrin-conjugated CD45 antibody (Figure 1D-F). The leukocytes adhering to the vessel appeared yellow in the merged images (Figure 1G-I).

The number of adherent leukocytes was significantly higher in STZ rats than in the control SD rats in the short term experiments (13.2±4.8 cells/retina vs 4.5±2.2 cells/retina, p<0.05) and in the long term experiments (21.2±2.2 cells/retina versus 6.5±3.0 cells/retina, p<0.05). These findings indicate that the number of adherent leukocytes, and thus the index of retinal leukostasis, increased significantly with the duration of diabetes (p<0.05; Figure 1J).

The SDT rats also showed a significant increase in the index of retinal leukostasis to that of controls in the short term experiments (12.0±3.0 cells/retina; p<0.05) and in the long term experiments (15.5±3.2 cells/retina; p<0.05), but the increase was not significant with the duration of diabetes (p<0.05). An important difference was that the index of retinal leukostasis was lower in SDT rats than in STZ rats in the long-term experiments (p<0.05, Figure 1J).

### Increased plasma pigment epithelium-derived factor expression in diabetic rats

**Figure 2:**
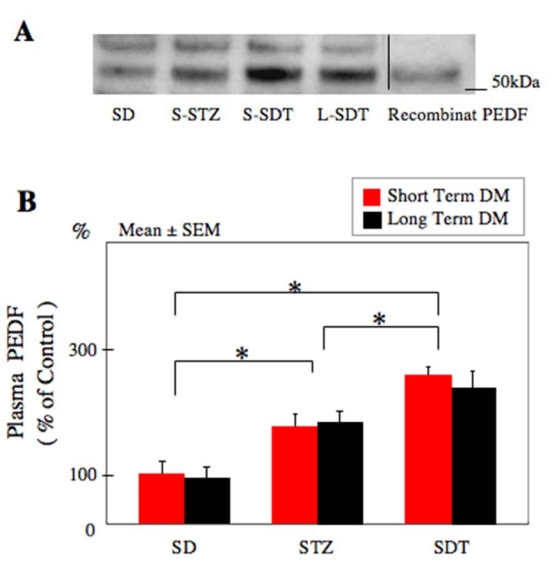
Western blot analysis for plasma pigment epithelium-derived factor expression. **A**: Data from 20-week-old control Sprague-Dawley (SD) rats, short term diabetes mellitus (DM)-streptozotocin-induced diabetic rats (S-STZ), short term DM-spontaneously diabetic Torii rats (S-SDT), and long term DM-SDT rats (L-SDT). The expression of pigment epithelium-derived factor (PEDF) was confirmed with the presence of recombinant PEDF (positive control, 50-kDa). **B**: Statistical analysis for the PEDF expression. PEDF expression is significantly higher in STZ rats in both the short term and long term experiments compared to that of SD rats. The expression of PEDF is also significantly higher in SDT rats compared to that of control (p<0.05). SDT rats had significantly higher levels of PEDF compared to that of STZ rats in both short term and long term diabetes mellitus. Data were analyzed by ANOVA with Fisher's LSD. Asterisk equals p<0.05. Eight eyes are used in each group at each study period.

When the expression of PEDF in the STZ and SDT rats was compared to that of controls, it was found there was a significant increase in PEDF in both STZ (172% increase in short term, 180% increase in long term; p<0.05, respectively) and SDT rats (264% increase in short term, and 239% increase in long term; p<0.05, respectively). PEDF levels were not significantly different between short term and long-term experiments in control, STZ, and SDT rats. An important difference was that the SDT rats had significantly higher levels of PEDF compared to that of STZ rats in both short term and long term diabetes (p<0.05 Figure 2).

### Increased plasma soluble intracellular adhesion molecule-1 levels in diabetic rats

**Figure 3:**
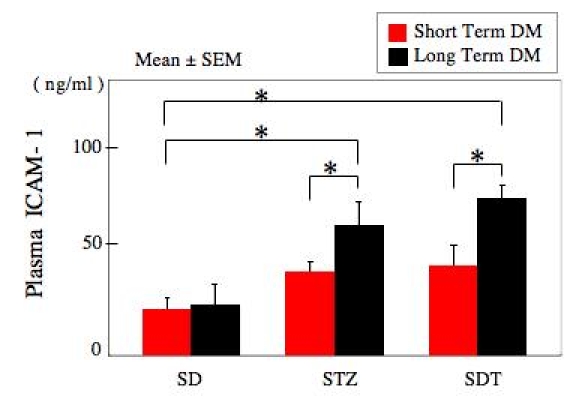
Plasma soluble intracellular adhesion molecule-1 levels. The levels of soluble intracellular adhesion molecule-1 (sICAM-1) were increased with duration of diabetes. The levels of sICAM-1 are significantly higher in long term both streptozotocin-induced diabetic (STZ) rats and spontaneously diabetic Torii (SDT) rats than in Sprague-Dawley (SD) rats (p<0.05), but the difference is not significant between short term diabetic rats and controls. In addition, there was not a significant difference in the level of sICAM-1 between the STZ and the SDT rats. The data were analyzed by ANOVA with Fisher's LSD. Asterisk indicates p<0.05. Eight eyes are used in each group at each study period.

In the long-term experiments, the level of sICAM-1 was significantly higher in both the STZ and SDT rats than in control SD rats (22.1±8.5 ng/ml, p<0.05). For the short term experiments, the level of sICAM-1 in the STZ and SDT diabetic rats was higher that that in the control SD rats, but the difference was not significant. In STZ rats, the sICAM-1 level was increased with an increase in the duration of diabetes (34.6±5.0 ng/ml in short term experiments versus 60.5±10.2 ng/ml in long term experiments, p<0.05). Similar findings were found in the SDT rats with a sICAM-1 level of 39.4±9.0 ng/ml in the short term experiments and 68.2±6.1 ng/ml in the long term experiments (p<0.05). However, there was not a significant difference in the level of sICAM-1 between the STZ and the SDT rats (Figure 3).

### Effects of pigment epithelium-derived factor and vascular endothelial growth factor on monocytic adhesion tohuman umbilical vein endothelial cells

**Figure 4:**
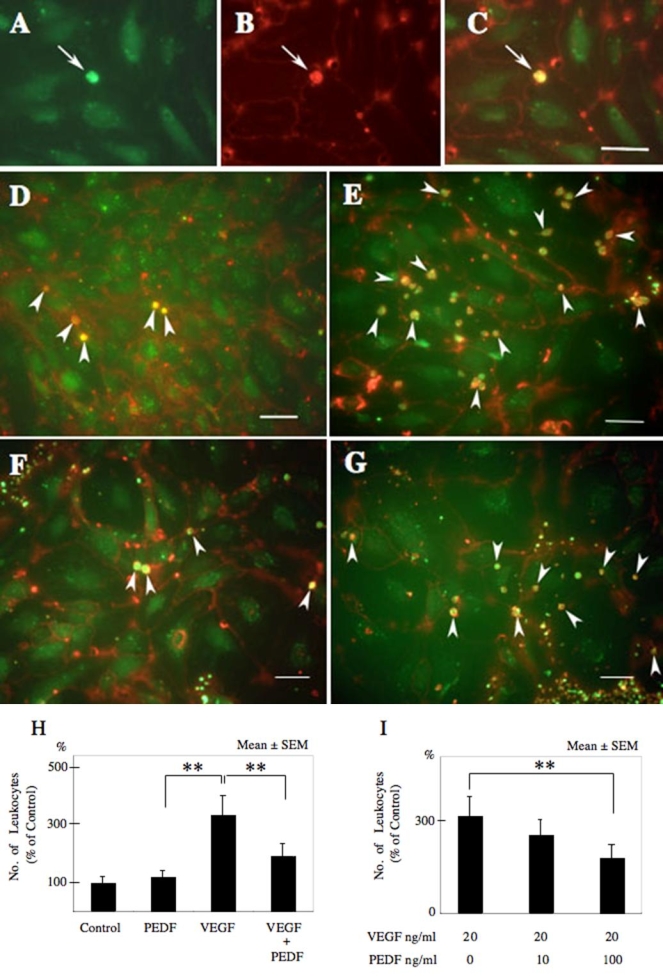
Confocal fluorescence microscopy for detection of adherent monocytes to human umbilical vein endothelial cells. Monocytes and nuclei of human umbilical vein endothelial cells (HUVECs) were labeled in green with Calcein-AM (**A**). The cell surfaces of the monocytes and HUVECs were labeled red with rhodamine-conjugated Con-A lectin (**B**). Adherent monocytes appeared yellow in the merged images obtained by combining FITC and rhodamine images (**C**). Arrows show monocytes. **D**-**G** show merged images of HUVECs and adherent monocytes. HUVECs were treated with phosphate buffered saline as a control (**D**), vascular endothelial growth factor (VEGF; **E**), pigment epithelial growth factor (PEDF; **F**), and both VEGF and PEDF (**G**). **H** shows quantification of adherent monocytes. Administration of PEDF did not significantly alter the number of adherent monocytes compared to that of controls. Alternatively, when VEGF was added, the number of adherent monocytes was significantly increased (p<0.01). On the other hands, when PEDF was jointly administered with VEGF, the increase of adherent monocytes induced by VEGF was significantly reduced (p<0.01). **I** shows the effects of PEDF on adherent monocytes induced by VEGF. PEDF appeared to inhibit the increase of adherent monocytes induced by VEGF in dose dependent manner. Results represent the average of eight experiments±SEM. The data were analyzed by ANOVA with Fisher's LSD. Double asterisks indicate p<0.01.

Monocytes and the nuclei of HUVECs were labeled green with calcein-AM (Figure 4A), and the cell surface of the monocytes and HUVECs were labeled red with rhodamine-conjugated Con-A lectin (Figure 4B). The adherent monocytes appeared yellow in the merged images obtained by combining the FITC and rhodamine images (Figure 4C).

The monocyte adhesion assay was performed on HUVECs treated with PBS as control (Figure 4D), with VEGF (20 ng/ml, Figure 4E), with PEDF (100 ng/ml, Figure 4F), and with PEDF + VEGF (Figure 4G) for 24 h. Exposure to PEDF did not significantly alter the number of monocytes adhering to HUVECs (119±24% increase) compared to that of controls. Alternatively, when VEGF was added to the media, the number of adherent monocytes was significantly increased (339±69%; p<0.01) compared to that of controls. When PEDF was added with VEGF, the number of adherent monocytes induced by VEGF dropped to 194±44% (p<0.01; n=8; Figure 4H).

When PEDF was combined with VEGF, PEDF appeared to inhibit the increase of adherent monocytes in a dose dependent manner (Figure 4I).

## Discussion

The adhesion of leukocytes to the retinal capillary endothelium has been shown to be an important event in animal models of diabetic retinopathy [11,22,23]. This adhesion plays a significant role in the breakdown of the blood-retinal barrier and in the occlusion of retinal capillaries [11]. ICAM-1 mediates the adhesion of neutrophils and monocytes to the vascular endothelium, and increased ICAM-1 levels have been found in the blood and in the retinal vasculature of patients with diabetes [17,24].

Recently, we reported that diabetic retinopathy in SDT rats is characterized by a low incidence of retinal neovascularization and an absence of retinal capillary occlusion [2]. The results of this study showed that leukostasis is increased in both SDT and STZ diabetic rats compared to that of controls. However, the index of leukostasis was significantly lower in SDT rats than in STZ rats in long-term experiments. STZ rats had a significant increase in the number of adherent leukocytes with longer durations of diabetes but SDT rats did not show this effect with the duration of the diabetes.

The plasma sICAM-1 levels were up-regulated in both SDT and STZ rats, and the increase was greater in long-term experiments. This is interesting because there was a significant difference in the index of leukostasis between STZ and SDT rats in the long tern experiments, although the sICAM-1 levels were not significantly different.

The plasma level of PEDF was increased in both SDT and STZ rats compared to that of controls, and the increase in the level of PEDF was significantly higher in SDT rats than in STZ rats. PEDF has been shown to be a potent inhibitor of angiogenesis [3]. The results of earlier studies have suggested that a decrease in the level of PEDF in the eye is involved in the pathogenesis of PDR in both animals and humans [4,5,8]. We have reported that the VEGF and PEDF levels in the retina of SDT rats were both up-regulated by Western blotting and immunohistochemistry [2]. Thus, higher levels of PEDF in the eye and in the blood most likely contributed to the lack of development of retinal neovascularization in SDT rats.

VEGF is a strong angiogenic factor and many previous studies have demonstrated its involvement in diabetic retinopathy [19]. VEGF can increase the expression of ICAM-1 on capillary endothelial cells in vitro and on the retinal vasculature in vivo [25,26]. Administration of PEDF has been shown to effectively inhibit retinal neovascularization and to reduce the VEGF-induced vascular hyperpermeability [7,27,28]. These results suggest that PEDF counteracts the effects of VEGF on retinal neovascularization. However, the mechanism for this inhibition has still not been determined. We hypothesized that the higher levels of PEDF in the plasma counteracted the effect of VEGF and also contributed to the low levels of leukostasis in SDT rats.

Our in vitro studies showed that VEGF increased the number of monocytes adherent to HUVECs, and this increase was significantly reduced by PEDF. In addition, PEDF also suppressed the VEGF-induced leukostasis in a dose-dependent manner, and the effective concentration (10-100 ng/ml) was lower than that for normal human subjects (5 μg/ml) [29]. We found that serum levels of VEGF were up-regulated in both SDT and STZ rats (unpublished data). VEGF-induced leukostasis is linked to capillary non-perfusion and increased vascular permeability [30]. Alternatively, PEDF can counteract VEGF-induced vascular permeability [28]. Although, retinal endothelial cells may have properties somewhat different from HUVECs, our results, together with the previous reports, indicated that PEDF is more likely to act as an inhibitor of VEGF function. Therefore, high levels of PEDF in the plasma of SDT rats might inhibit the VEGF-induced leukostasis and result in the absence of capillary occlusion and lower incidence of retinal neovascularization. Additional studies are needed to determine the molecular mechanism by which PEDF inhibits VEGF.

PEDF can reduce VEGF activity and VEGF induced leukostasis, but we found increased levels of sICAM-1 in SDT rats. It is possible that PEDF may also directly counteract the activity of ICAM-1. In diabetic retinas, when the VEGF bioactivity is blocked, the up-regulation of ICAM-1, leukocyte adhesion, and BRB breakdown are substantially reduced [19]. Chronic, low-grade inflammation is responsible for many of the signature vascular lesions of diabetic retinopathy [18]. PEDF has been suggested to be an anti-inflammatory cytokine [31], thus, high levels of PEDF in the retina and plasma may inhibit inflammation in diabetic retinas.

The accumulation of advanced glycation end products (AGEs) in the diabetic retina is also involved in retinal leukostasis and microthrombosis by enhancing the expression of adhesion molecules [32,33], inducing apoptosis of pericytes, and up-regulating VEGF [34]. These changes then contribute to focal ischemia in early diabetic retinopathy. Recently, it was reported that PEDF prevented diabetes- or AGE-elicited retinal leukostasis [35] and also inhibited AGE-induced cell death of pericytes [36] and monocyte chemoattractant protein-1 production in microvascular endothelial cells [37]. These activities of PEDF on AGE may also contribute to reduction of leukostasis.

In conclusion, PEDF inhibits VEGF-induced leukostasis in vitro, and this action most likely explains the low incidence of capillary occlusion and retinal neovascularization in SDT rats. These findings suggest that PEDF should be examined as a possible therapeutic agent for the treatment of diabetic retinopathy.
